# Cell-free DNA and circulating tumor cell kinetics in a pre-clinical head and neck Cancer model undergoing radiation therapy

**DOI:** 10.1186/s12885-021-08791-8

**Published:** 2021-10-02

**Authors:** Nidal Muhanna, Donovan Eu, Harley H. L. Chan, Catriona Douglas, Jason L. Townson, Marco A. Di Grappa, Reza M. Mohamadi, Shana O. Kelley, Scott V. Bratman, Jonathan C. Irish

**Affiliations:** 1 Princess Margaret Cancer Center, University Health Network, Toronto, ON Canada; 2 TECHNA Institute, Guided Therapeutic (GTx) Program, University Health Network, Toronto, ON Canada; 3 Department of Otolaryngology-Head and Neck Surgery-Surgical Oncology, University of Toronto, Toronto, Ontario Canada; 4 Department of Otolaryngology-Head and Neck Surgery, Tel Aviv Sourasky Medical Center, Tel Aviv University, Tel Aviv, Israel; 5 Leslie Dan Faculty of Pharmacy, University of Toronto, Toronto, ON Canada; 6 Department of Radiation Oncology, University of Toronto, Toronto, ON Canada

**Keywords:** Circulating tumor cell, Circulating tumor DNA, VX2, Rabbit, Radiation, Head and neck cancer, Oral cavity cancer preclinical model

## Abstract

**Background:**

Monitoring circulating tumor DNA (ctDNA) and circulating tumor cells (CTCs), known as liquid biopsies, continue to be developed as diagnostic and prognostic markers for a wide variety of cancer indications, mainly due to their minimally invasive nature and ability to offer a wide range of phenotypic and genetic information. While liquid biopsies maintain significant promising benefits, there is still limited information regarding the kinetics of ctDNA and CTCs following radiation therapy which remains a vital treatment modality in head and neck cancers. This study aims to describe the kinetics of ctDNA and CTCs following radiation exposure in a preclinical rabbit model with VX2 induced buccal carcinoma.

**Methods:**

Seven rabbits were inoculated with VX2 cells in the buccal mucosa and subjected to radiation. At selected time points, blood sampling was performed to monitor differing levels of ctDNA and CTC. Plasma ctDNA was measured with quantitative PCR for papillomavirus E6 while CTCs were quantified using an immunomagnetic nanoparticles within a microfluidic device. Comparisons of CTC detection with EpCAM compared to multiple surface markers (EGFR, HER2 and PSMA) was evaluated and correlated with the tumor size.

**Results:**

Plasma ctDNA reflects the overall tumor burden within the animal model. Analysis of correlations between ctDNA with tumor and lymph node volumes showed a positive correlation (R = 0.452 and R = 0.433 [*p* < 0.05]), respectively. Over the course of treatment, ctDNA levels declined and quickly becomes undetectable following tumor eradication. While during the course of treatment, ctDNA levels were noted to rise particularly upon initiation of radiation following scheduled treatment breaks. Levels of CTCs were observed to increase 1 week following inoculation of tumor to the primary site. For CTC detection, the use of multiple surface markers showed a greater sensitivity when compared to detection using only EpCAM. Plasma CTC levels remained elevated following radiation therapy which may account for an increased shedding of CTCs following radiation.

**Conclusion:**

This study demonstrates the utility of ctDNA and CTCs detection in response to radiation treatment in a preclinical head and neck model, allowing for better understanding of liquid biopsy applications in both clinical practice and research development.

## Background

The use of liquid biopsies for cancer management has continued to attract increasing research focus during the past decade. The potential to detect cancer using simple, minimally invasive blood sampling could allow for improved screening, diagnosis, prognosis and monitoring of therapeutic response and in certain cases decreased morbidity [1–3]. Furthermore, with the paradigm shift in personalized medicine, precision oncology is currently in development for treatment of specific cancers based on genomic markers and proteomic expression within the cancer, which may be readily discerned via liquid biopsies [4–6]. Since the descriptions of circulating tumor cells in 1869 and cell free DNA 1948, there has been a multitude of studies that have detailed the potential advantages of liquid biopsies in clinical application [7, 8]. Liquid biopsies in head and neck cancers have been shown to have a potential role in early diagnosis in nasopharyngeal cancers using Epstein-Barr virus DNA while combination of saliva and plasma ctDNA shows promise for early detection of mucosal head and neck squamous cell carcinomas [9–11]. However, the lack of prospective clinical trials and lack of studies demonstrating conclusive clinical validity have limited their roles in routine clinical practice at this time [12].

Two prominent methods of attaining a liquid biopsy include the detection of circulating tumor cells (CTC) and circulating DNA (ctDNA). The presence of CTCs is thought to act in part as a marker of potential metastasis, by indicating their intravasation into the circulatory system from the primary tumor. Following intravasation, they may then metastasize into local or distant tissues [13, 14]. As the tumor evolves, epithelial-mesenchymal transition is hypothesized to lead to the loss of epithelial characteristics, thereby diminishing the levels of CTCs detected using epithelial markers. This is particularly evident with epithelial cell adhesion molecules (EpCAMs), a well characterized CTC marker [15]. Thus utility of EpCAM as the CTC capture agent may in part demonstrate the progression of tumor and possible de-differentiation which carries significance especially during the course of treatment. ctDNA on the other hand consists of short DNA fragments released into the circulation following cell death and turnover that has shown good correlation with tumor burden in several solid organ tumors [16, 17].

While these techniques show promise, their limitations such as inter-laboratory variability in processing and interpretation have prevented them from mainstream clinical use. In addition, there is also a lack of studies on the effects of radiation on ctDNA and CTCs. This has relevance for patients with head and neck malignancies, where a substantial proportion of patients are treated with primary radiation or chemo-radiation. It is apparent that more studies are required before translation into clinical use.

With our prior work in kinetics of ctDNA following surgery as well as a novel nanoparticle based detection of CTC in a preclinical model, we sought to develop a similar model to determine the kinetics of CTC and ctDNA in response to radiation therapy [18, 19]. The establishment of such a preclinical model will allow for greater understanding of the kinetics of liquid biopsies in response to radiation, which may be of significance as a biomarker in further development of combination therapy.

## Methods

### Animal model

Experiments were performed using male New Zealand white rabbits obtained from Charles River Institution (Wilmington, Massachusetts), weighing 2.5–3.0 kg in accordance with the University Health Network/University of Toronto guidelines for the humane use of animal care. This study was reviewed and approved by the ethics committee under the Animal Resource Centre, University Health Network, University of Toronto under Animal Utilization Protocol #2931.19. A total of 7 rabbits were involved in this study. Rabbits were naïve to previous treatment / experiments and were assessed by the veterinarians prior to the study to ensure fitness. No inclusion/exclusion criteria were used for selection of these rabbits. Rabbits were individually caged in climate-controlled rooms with a 12-h light cycle and provided with food and water ad libitum and allowed to acclimatize to facilities for at least 7 days prior to experimental start date. The care and maintenance of these animals were performed in a humane manner with strict compliance to the animal care experimental protocol approved by the institutional animal care and use committee of the University Health Network, University of Toronto.

To induce VX2 buccal tumors, 1cm^3^ of VX2 tumor was passed through a 100 μm filter to create a tumor cells suspension using PBS. Seven rabbits were injected with the cell suspension of VX2 squamous cell carcinoma into the right buccal mucosa. Tumor progression was evaluated by clinical examination twice a week and imaging performed weekly. Rabbits were clearly marked via marker pen along the ear and separate dates scheduled for monitoring, imaging and treatment to minimize confounders. At endpoint, rabbits were euthanized following the approved protocol, consisting of anesthesia using isofluorane followed by overdose with potassium chloride. Outcomes for this study involved the quantification of ctDNA and CTC from serial serological evaluation following tumor induction and radiation treatment. No animals were used as a control group, as this study was a performed to demonstrate proof of concept. All data attained from the animals was included in the final analysis.

### CT imaging and image analysis

CT imaging was performed at day 5 post VX2 cell injection to ascertain the presence of a viable tumor. Thereafter repeat CT was repeated on the first, third and fifth day of radiation of each cycle. Following completion of the planned course of radiation, imaging was acquired weekly thereafter. CT imaging (Locus Ultra, GE Healthcare, Milwaukee, Wisconsin, USA) was performed weekly (80 kVp, 50 mA). CT image analysis was performed using Microview (GE Healthcare, Milwaukee, Wisconsin, USA) and custom in-house program written using MATLAB (MathWorks®, Natick, Massachusetts, USA). The tumor volumes were contoured using a semi-automated threshold-based method, the mean and standard deviation of the voxel signal distribution within each VOI were calculated.

### Radiotherapy intervention

#### Radiation for ctDNA

**Fig. 1:**
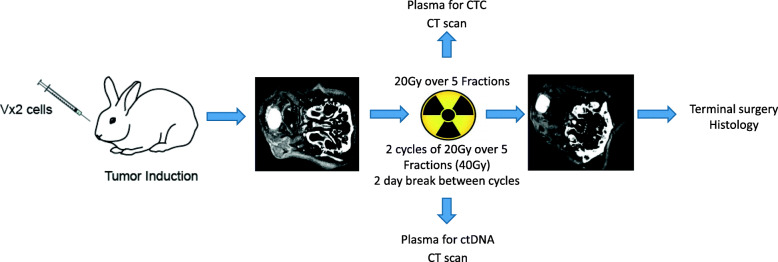
Diagrammatic representation of study protocol. Rabbits were injected with VX2 cells into the buccal mucosa and tumor growth confirmed on CT on day 5 following injection. Two cycles of 20Gy over 5 fractions of photon radiation was administered daily with a 2 day treatment break in between each cycle for rabbits undergoing ctDNA evaluation while a single cycle of 20Gy over 5 fractions was administered to the rabbits undergoing CTC evaluation. Serology was obtained at specified time points. All rabbits were sacrificed for formal histology correlation

Four rabbits underwent primary radiation when tumor was approximately 1 cm in size. This usually occurred 10 days after inoculation of tumor. They each received two cycles of 4Gy for 5 days (40Gy total) with a treatment break of 2 days between the first and second cycle (Fig. 1). Treatment breaks over the weekend was designed in compatibility to standard radiation treatment in most centers. The decision for early radiation at day 10 was made to mimic early disease that can be treated with unimodality radiation treatment. Furthermore, it has been noted that the presence of ctDNA fluctuates with tumor necrosis, which for the purpose of this study obscures the effects of radiation. Blood was taken for analysis on days of radiation and selected time points after radiation. On days where radiation treatment was planned, all blood samples were acquired prior to the radiation procedure. In summary, blood taking was performed at days 10–15, 17–21, 25, 27, 31 after tumor injection. The rabbits were euthanized by isoflurane anesthesia followed by KCl injection (125 mg/kg IV) on day 31 and histological sections obtained.

#### Radiation for CTC

Three rabbits underwent primary radiation approximately 17 days after inoculation of tumor. All rabbits received a total of 20Gy (4Gy per fraction, one fraction a day for 5 days); no treatment breaks were allocated. These rabbits were monitored by imaging in the form of CT scans and blood sampling via the ear veins for CTCs before radiation and every other day during and following radiation. These rabbits underwent radiation at a later date compared to the rabbits from the ctDNA cohort, in view of the greater tumor volume necessary to detect CTCs. Rabbits were euthanized (isoflurane followed by 125 mg/kg IV KCl) 3 days after completion of radiation treatment, imaging was acquired prior to sacrifice.

### Quantification of ctDNA

The VX2 tumor model is associated with the cottontail rabbit papillomavirus (CRPV). This is similar to the human papilloma virus (HPV) which has been established as a key oncovirus for cancers of the oropharynx. Thus, this model closely resembles the management of a patient with HPV derived oropharynx cancer. The target for ctDNA detection utilizes primer probes for oncogene E6 or E7. For the purpose of this study, plasma ctDNA was detected by means of a previously validated qPCR assay based on a 58-bp E6 assay derived from the E6 open reading frame (ORF) [18]. From prior studies, the 58-bp E6 assay has shown to be sensitive enough to achieve robust detection of CRPV DNA sequences form as little as 3.7 pg of VX2 genomic DNA. Specificities for this test also remain high showing no evidence in samples of rabbits that were not inoculated with VX2. In addition to ctDNA, the collection of cell-free DNA (cfDNA) and total DNA in the serum was collected for further analysis.

### Quantification of CTC

**Fig. 2:**
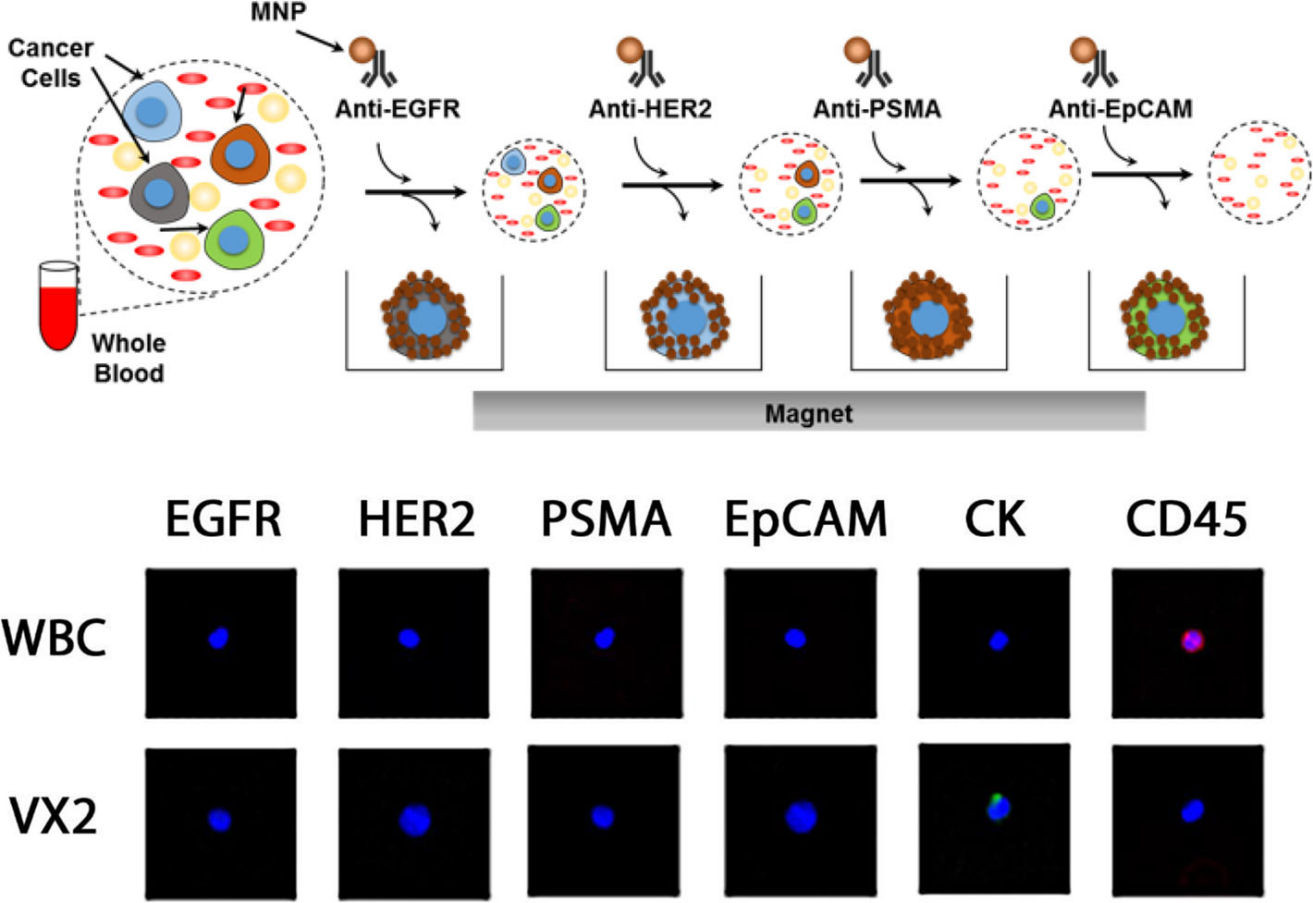
Diagram above demonstrates a schematic of the action of TxViva in detection of EGFR, HER2, PSMA, EPCAM, CK and CD45. Micrograph below demonstrate CTC clusters isolated of the above markers with TxViva comparing VX2 and WBC

CTC quantification was performed using immunomagnetic nanoparticles within a microfluidic device that has been previously described [19]. In summary, blood taken from the rabbit was incubated with MACS anti-CTC nanoparticles for 10 min. Thereafter, this blood was then introduced into a fabricated microchip and PBS-EDTA used to rinse out red blood cells before fixation with 100% formaldehyde. Following this, immunostaining was performed via CTC-specific antibodies. This method of CTC measurement, which will be referred to as TxViva, was preferred due to the ability to distinguish the differential expression of these epithelial markers (Fig. 2). In addition, it also allows for capture of multiple surface markers, such as Epithelial Growth Factor Receptor (EGFR), Human Epidermal Growth Factor 2 (HER-2), Prostate Specific Membrane Antigen (PSMA) and Epithelial Cell Adhesion Molecule (Epcam) (Fig. 1). Comparisons of CTC detection was performed between EpCAM alone and TxViva from a 2 ml blood sample.

### Statistical analysis

Analysis of correlation between Plasma ctDNA and overall tumor burden by means of tumor and lymph node volumes were assessed by Pearson’s correlation. Statistical analysis was performed using GraphPad Prism 7 (GraphPad Software, La Jolla, CA, USA). A *P*-value of < 0.05 was considered significant.

## Results

### Analysis of circulating tumor DNA

**Fig. 3:**
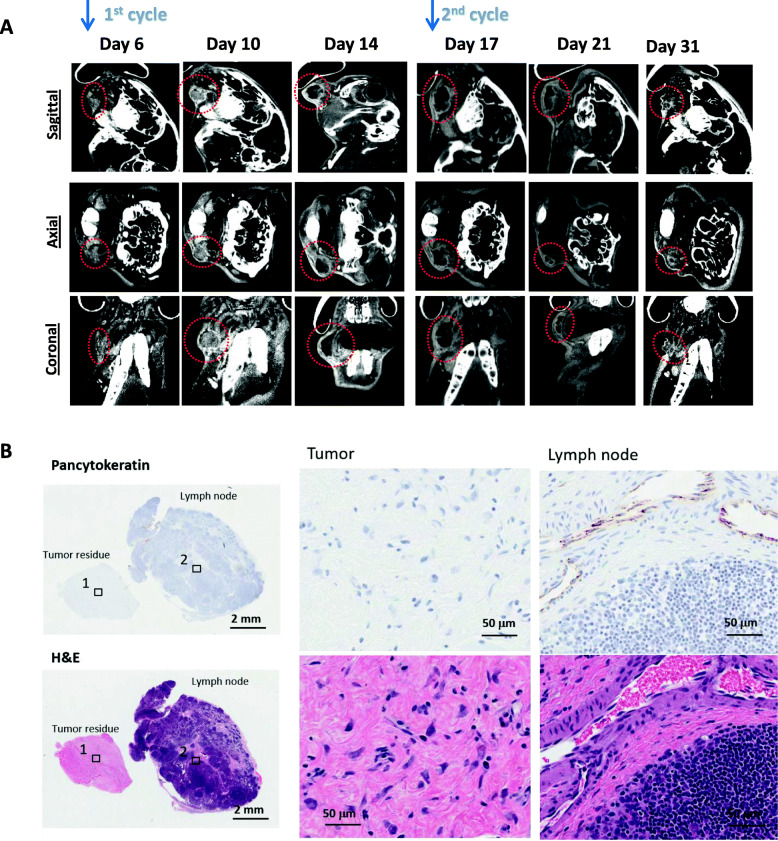
A) CT performed at different time points demonstrating the tumor size (indicated by dotted red lines) with correlation of the radiation cycles. B) Histological slides (H&E and pancytokeratin stains) demonstrating eradication of tumor at low and high magnification

For this cohort of rabbits, the mean tumor volume was measured at 1332 mm^3^, and the median volume was 1334 mm^3^. Tumor volumes were obtained by CT imaging and correlated with radiation time points (Fig. 3A). Viable and non-viable tumor components were distinguished on CT based degree of hypodensity measured by Hounsfield units. The mean viable and non-viable tumor volumes were 659 mm^3^ and 575 mm^3^, respectively.

**Fig. 4:**
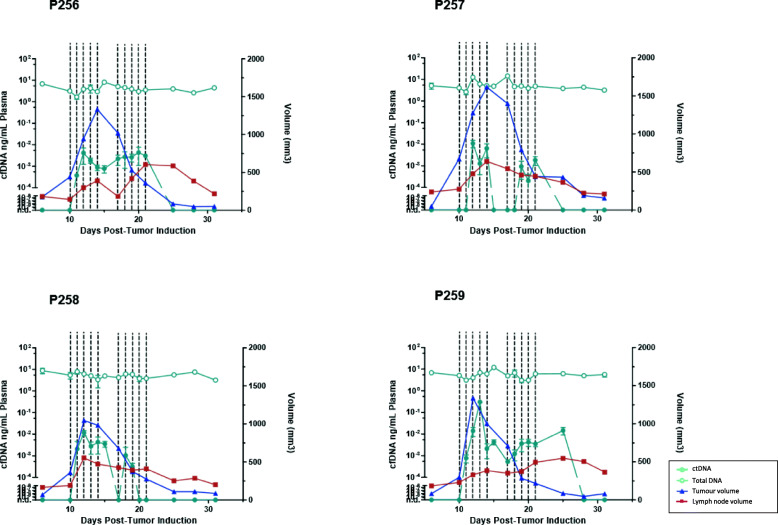
Demonstrates the fluctuations of ctDNA with respect to tumor and nodal volumes during the course of the experiment. The vertical dotted lines indicate the days that rabbits underwent radiation. Plasma ctDNA shows a good correlation with primary tumor volumes and mirrors closely the pattern of tumor volume. Plasma ctDNA elevation is noted during the initiation of radiation in both first and second cycles

**Fig. 5:**
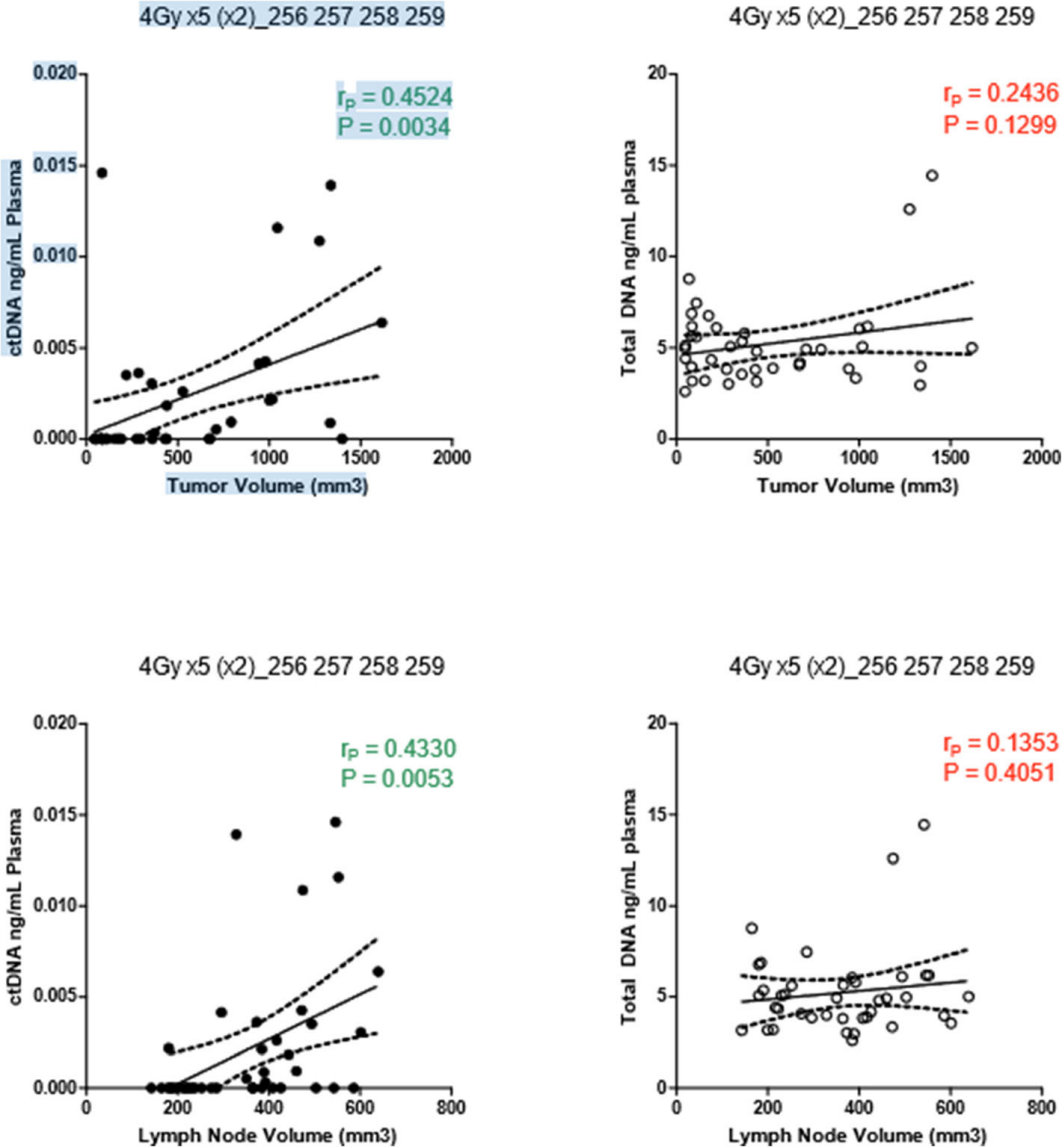
Scatter plot showing correlation between ctDNA and total tumor and lymph node volumes. Plasma ctDNA shows good correlation (Pearson r) with both primary tumor volumes as well as lymph node volumes. Total DNA however failed to show a significant correlation between tumor and lymph node volumes

ctDNA remained undetectable until day 10 post-inoculation when tumor volume approached an average of 440 mm^3^. In the majority of the rabbits, detection of ctDNA was noticeable on day 11. During the course of radiation, ctDNA levels (Fig. 4) were noted to mirror the total tumor volumes. Further analysis of the association between ctDNA with tumor and lymph node volumes showed a positive correlation (R = 0.452 and R = 0.433 [*p* < 0.05]), respectively (Fig. 5). In contrast, measurements of total plasma cell-free DNA (cfDNA) did not show any correlation to either tumor or nodal volumes. Following treatment, a decline in ctDNA further correlated the sharp drop in tumor volumes in all 4 rabbits. Additionally, an elevation in ctDNA was notable at the 2nd cycle of radiation – following the 2 day treatment break. In all but 1 rabbit, ctDNA levels fell to undetectable range following final radiation treatment on day 21. This in turn correlated to decrease in both tumor and lymph node volumes. Failure of ctDNA to become undetectable in that single rabbit was attributed to regional metastasis to the cervical lymph node, which was identified on imaging. Final histological evaluation of this cohort of rabbits showed no evidence of tumor in both primary site and nodal basin (Fig. 3B).

### Circulating tumor cell capture using TxViva and EpCAM systems

**Fig. 6:**
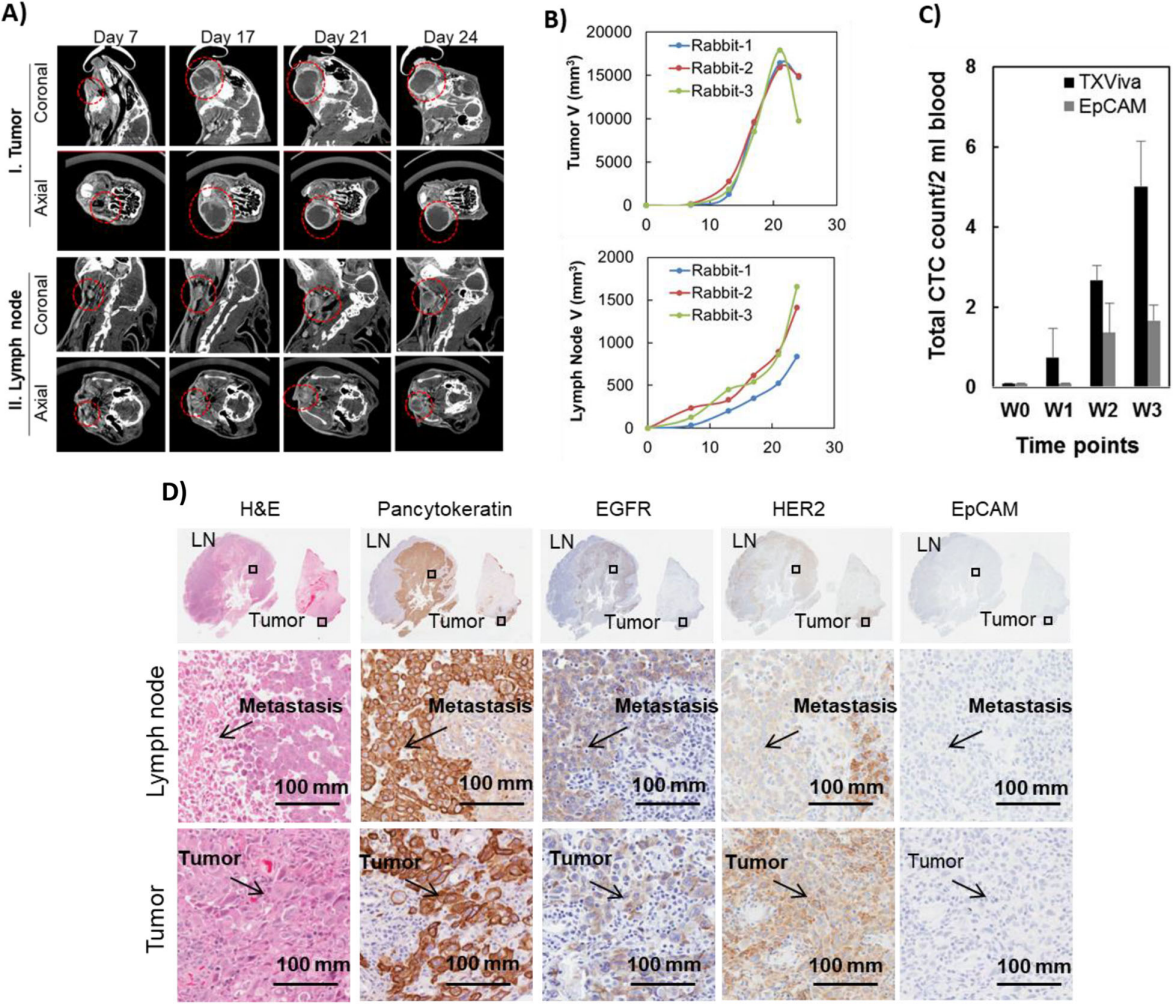
A) CT images showing tumor growth and nodal disease (marked by dotted red lines) at selected time points. B) Volumetric assessment of primary tumor and lymph nodes. C) CTC levels at weekly timepoints using TxViva and Epcam. D) Histological analysis of tumor and lymph nodes using H&E, pancytokeratin, EGFR, HER2 and Epcam demonstrating residual tumor in both primary and lymph nodes

At day 17 following inoculation, the primary tumors reached a size of 15,000 mm^3^ (Fig. 6A and B). The mean size of tumor was 16,749 mm^3^ and median volume was 16,421 mm^3^. TxViva CTC detection (capture via anti-EGFR, anti-HER2, anti-PSMA, anti-EpCAM) demonstrated increased sensitivity when compared to identification of Epcam alone (Fig. 6C). Levels of CTC was notably elevated as early as week 1 after inoculation when using TxViva, while EpCAM detection alone was only noticed on week 2 of inoculation. In all time points between week 1 to 3, TxViva demonstrated higher levels of CTC detection when compared to detection by EpCAM alone.

## Discussion

Liquid biopsies using ctDNA and CTC have been demonstrated to have potential for a diverse range of diagnostic, prognostic and treatment response monitoring applications. In the clinic, this could allow for an additional biochemical monitoring for patients who are at risk of treatment failure. For the purpose of research and treatment development, these markers are valuable to distinguish treatment accuracy and validate target acquisition particularly with regards to radiation therapy. However, a full understanding of the behavior of these biomarkers with respect to radiation treatment remains to be obtained [20]. A preclinical model has the advantage of being able to control variables to allow for a homogenous cohort, which is essential to effectively demonstrate the effects of radiation on the levels of ctDNA and CTC.

As evident in our study, CTC detection, especially when only EpCAM capture was utilized, was only feasible when tumors were larger and more advanced in contrast to ctDNA. This is not surprising, as CTCs are thought to be harbingers of metastatic deposits in the “seed and soil” theory. Early and reliable detection has a significant utility to determine the aggressive nature of the primary tumor that may aid in treatment decisions. Our experience with CTC for the monitoring of tumor growth in this head and neck model shows an increased sensitivity when utilizing TxViva as a detection method in comparison to EpCAM only capture method. This is noticeable via earlier detection of rare cells, such as EpCAM, Her-2, PSMA and EGFR in the circulation as early as week 1 following inoculation. Elevations of CTC in weeks 2 and 3 further demonstrated a higher sensitivity profile compared to EpCAM only CTC capture methods.

To date, Cellsearch (which uses predominantly EpCAM detection) is the only FDA approved method of CTC enumeration and its sensitivity in detection of CTC has been challenged in several other publications. Techniques that incorporate other microfluidic systems or the use of isolation via size of epithelial tumor cells (ISET) filtration methods have been explored to improve detection of CTC. Direct comparisons between ISET and Cellsearch has shown a greater detection rate in other sites such as hepatocellular carcinoma, pancreatic cancer and non-small cell lung cancers [21–23]. In addition to ISET, there have been several other microfluidic chips such as spiral microfluidics and ClearCell Fx System that have also been evaluated for head and neck cancers [24, 25].

Clinically, the identification of CTC has gained significant attention over the past decades. It is hypothesized that CTC may be useful to determine radioresistance and further aid in prognostication of treatment outcomes. However, there is yet to be a reliable method for sensitive detection of these rare cells. Our results here show similar findings with the current literature in the limitations in sensitivity of CTC detection by Cellsearch.

For the purpose of ctDNA detection, we created a design for the treatment of early head and neck cancers using radiation as a single modality treatment. Each animal underwent a total of 40 Gy over 10 fractions, with a 2-day treatment break in the middle of treatment. This multi-fraction approach mimics current standard treatment of early head and neck cancers. As demonstrated in our results, there remains a good correlation between levels of ctDNA with overall tumor burden (Fig. 4A). In the context of early primary tumor, levels of ctDNA closely mirrored the primary tumor volumes in all but 1 rabbit. This was thought to be attributed to nodal metastasis, which corresponds to the transient increase in nodal volume despite the dramatic decrease in size in the tumor volumes. Despite this being an early stage tumor, it is established that up to 20–30% of early staged tumors may be associated with occult nodal metastasis [26]. This would be consistent with our study cohort. Furthermore, the subsequent drop in ctDNA also corresponds to the decline in nodal volumes. We postulate that this may represent an abscopal effect of radiation which can explain the drop in tumor burden and hence the decline in ctDNA. Final histology (Fig. 3B) at 10 days following completion of radiation similarly shows no residual tumor in both primary site and lymph node.

Following treatment with radiation, plasma ctDNA was noted to begin its decline towards the third to fourth day of radiation treatment. Interestingly, all the rabbits demonstrated an acute elevation in plasma ctDNA at the beginning of the second cycle of radiation. It is our interpretation that this sudden surge of plasma ctDNA is likely from acute release of tumor DNA into the circulation following local tumor destruction. It is possible that this is potentiated after the 2 day treatment break due to the influx of macrophages and subsequent increase in phagocytosis, or possible restoration of vasculature within the tumor both of which are essential for tumor DNA’s escape into the circulation [27]. Plasma ctDNA levels however quickly declines following completion of treatment, this is in contrast to imaging which often shows indeterminate features within the treated fields. This is reinforced with histological evidence of complete tumor resolution despite imaging features. In that aspect, ctDNA allows for greater accuracy in predicting complete tumor resolution compared to imaging and allows for a noninvasive method to establish complete clinical response in the early period following radiation treatment.

Plasma ctDNA proves to be a viable biomarker of tumor volume within this model. For the purpose of head and neck research, we postulate this may be of value to assess biological response of treatment within preclinical trials involving combination therapies and future radiation modalities. As this is an immunocompetent model, the possibilities of utilizing this model for combination therapy with immunotherapy and radiation guided by ctDNA levels makes for an interesting avenue to be explored.

From our study, plasma ctDNA declines rapidly following tumor eradication. As such, it may represent a more reliable determinant of complete therapeutic response and may be used as a surrogate in lieu of early post-treatment imaging. Plasma ctDNA may also allow for confirmation of target acquisition during radiation treatment, as evident by the surge of ctDNA seen during initiation of the second cycle. This marker may be further utilized in the context of advanced radiotherapy modalities, as it provides biologic feedback that confirms an on-target response following precision radiotherapy.

Liquid biopsies in the form of CTC and ctDNA have been proposed as an adjunct resource for cancer monitoring. These cancer markers allow monitor of disease activity on the biological front, thereby potentially alerting the clinicians to recurrent/residual disease prior to structural evidence that is picked up by imaging. Early studies have shown the utility of CTC detection in the setting of head and neck cancers in predicting pulmonary metastasis and has been shown prognostic values in patients with recurrent or metastatic head and neck cancers [28, 29]. The value of ctDNA in the setting of EBV related nasopharyngeal cancers and HPV derived oropharyngeal cancers further show great promise in some clinical trials. EBV DNA has shown value for prognostication to treatment response and preclinical detection of tumor recurrence, while detection of plasma HPV DNA has also been successful in assessment of tumor recurrence in oropharyngeal cancers [30–32]. These studies show the utility of liquid biopsies in patient care following radiation treatment. As such, better understanding of CTCs and ctDNA in the peri-treatment period is important to comprehend and correlate its post-treatment effects.

From this series of 7 animals, we attempted to evaluate the kinetics of both CTC and ctDNA in their response to radiation treatment. Certainly, there are limitations in drawing conclusions form such a small sample size however, as the aim was to achieve a qualitative analysis of liquid biopsy response to radiation, it was felt that a small cohort of animals would suffice. From our study conduct, it is clear that rabbits evaluated by ctDNA and CTC had different tumor sizes and different treatment regimes. This limitation was largely due to the need for greater tumor progression before CTC could be evaluated in the blood. As these tumors were significantly larger, these rabbits had a shorter follow-up period as they reached humane endpoints earlier than the ctDNA cohort.

## Conclusion

Liquid biopsies remain a promising method to monitor tumor progression and response to treatment. We describe here the use of a medium-sized immunocompetent animal model and demonstrate proof of concept application with the use of liquid biopsies following radiation treatment of head and neck cancer models. With regards to CTC, the use of TxViva which encompasses detection of 4 surface markers show a greater sensitivity in comparison to EpCAM only capture. For the purpose of radiation treatment, ctDNA shows good overall correlation to tumor volumes and further demonstrates intermittent spikes in levels during radiation exposure which may possibly be utilized to confirm an on-target biochemical response. Our findings here further demonstrate liquid biopsies may be a useful clinical tool necessitating further preclinical evaluation and development. Ultimately, clinical studies will be required to more thoroughly evaluate and develop the utility of this minimally invasive method for measuring systemic response of treatment for head and neck cancers. Such development for clinical application could provide several benefits including providing a non-ionizing, minimally invasive method of monitoring therapeutic efficacy, residual and systemic disease and recurrence.

## Data Availability

The datasets used and/or analysed during the current study are available from the corresponding author on reasonable request.

## Abbreviations

ctDNA: Circulating DNA
CTC: Circulating tumor cells
PCR: Polymerase chain reaction
EpCAM: Epithelial cell adhesion molecule
EGFR: Epithelial growth factor receptor
HER2: Human epidermal growth factor 2
PSMA: Prostate specific membrane antigen
ORF: Open reading frame
